# An Exploration of Factors Linked to Academic Performance in PISA 2018 Through Data Mining Techniques

**DOI:** 10.3389/fpsyg.2020.575167

**Published:** 2020-11-27

**Authors:** Adriana Gamazo, Fernando Martínez-Abad

**Affiliations:** Research Institute on Educational Sciences, University of Salamanca, Salamanca, Spain

**Keywords:** educational data mining, school performance, large-scale assessment, non-cognitive outcomes, socioeconomic status, decision tree, academic achievement

## Abstract

International large-scale assessments, such as PISA, provide structured and static data. However, due to its extensive databases, several researchers place it as a reference in Big Data in Education. With the goal of exploring which factors at country, school and student level have a higher relevance in predicting student performance, this paper proposes an Educational Data Mining approach to detect and analyze factors linked to academic performance. To this end, we conducted a secondary data analysis and built decision trees (C4.5 algorithm) to obtain a predictive model of school performance. Specifically, we selected as predictor variables a set of socioeconomic, process and outcome variables from PISA 2018 and other sources (World Bank, 2020). Since the unit of analysis were schools from all the countries included in PISA 2018 (*n* = 21,903), student and teacher predictor variables were imputed to the school database. Based on the available student performance scores in Reading, Math, and Science, we applied k-means clustering to obtain a categorized (three categories) target variable of global school performance. Results show the existence of two main branches in the decision tree, split according to the schools’ mean socioeconomic status (SES). While performance in high-SES schools is influenced by educational factors such as metacognitive strategies or achievement motivation, performance in low-SES schools is affected in greater measure by country-level socioeconomic indicators such as GDP, and individual educational indicators are relegated to a secondary level. Since these evidences are in line and delve into previous research, this work concludes by analyzing its potential contribution to support the decision making processes regarding educational policies.

## Introduction

The emergence of international large-scale assessments (ILSA) in the past two decades, together with their cyclic nature, have consistently provided educational researchers with large databases containing diverse types of variables (student performance and background, school practices and processes, etc.). Assessment schemes such as the Programme for International Student Assessment (PISA) from the Organisation for Cooperation and Economic Development (OECD), or the Trends in International Mathematics and Science Study (TIMSS) and the Progress in International Reading Literacy Study (PIRLS), both conducted by the International Association for the Evaluation of Educational Achievement (IEA), have had a noticeable impact on the development of educational research in past years (Gamazo et al., 2016). But the great relevance of large-scale assessments is not circumscribed to educational research; some authors also highlight the great impact that PISA results have on national policies and practices in the field of education (Lingard et al., 2013). However, it has been observed that educational policies are usually influenced by the reports and analyses elaborated directly by the OECD, because these are the first ones presented to the public after a given PISA wave (Wiseman, 2013) and since these analyses can be somewhat limited considering the vast array of variables that PISA offers (Jornet, 2016), there is a certain responsibility for educational researchers to delve deeper into the databases and find relationships among variables and conclusions that might not be offered by the OECD reports in order to enrich the political debate around the topic.

Secondary analyses of PISA data can be performed through the use of different methodologies. One of the most common ones is multilevel regression analysis, given that it allows researchers to account for the variability at the level of students and schools at the same time (Willms, 2010; Gamazo et al., 2018). Other authors have opted for different methods, such as Structural Equation Modeling (Acosta and Hsu, 2014; Barnard-Brak et al., 2018) or ANCOVA (Smith et al., 2018; Zhu and Kaiser, 2019). Additionally, thanks to the emergence of big data, new possibilities in the statistical analysis of all types of databases have appeared in recent years. Namely, data mining has appeared in the past few years as one of the emerging techniques to analyse PISA data (Liu and Whitford, 2011; Tourón et al., 2018; Martínez-Abad, 2019; She et al., 2019), although it is a less-explored analysis method.

The data mining approach seeks to detect key information in huge amounts of data (Witten et al., 2016). Thus, data mining algorithms are specifically defined to be used in extensive databases, like those from large-scale assessments. These kinds of techniques build and validate models directly from the empirical data, without the use of either theoretical distributions or hypothesis tests (Xu, 2005), and allow the joint inclusion of both categorical and numerical variables. That is why, unlike the inferential and multivariate approaches, the models obtained through data mining algorithms are inductive, that is, computed exclusively from the information contained in the database. This way, data mining techniques can help to identify the main factors linked to academic performance and its interactions under a new framework, allowing researchers to reassess and refine existing theoretical models.

However, it is worth noting that the power of data mining resides in the production of exploratory studies to identify potentially significant relationships within large amounts of data, but follow-up confirmatory studies would be necessary in order to consolidate findings (Liu and Ruiz, 2008). Additionally, data mining presents other weaknesses that researchers must take into consideration, such as possible misinterpretations due to human judgment on the findings, an information overload leading to the construction of highly complex relationship systems, or the difficulty to interpret data mining results on the part of educational professionals (Papamitsiou and Economides, 2014).

Thus, the main aim of this paper is to take advantage of the benefits offered by data mining techniques in order to explore the influence of different types of student, school, and country variables on student performance in reading, science and mathematics in PISA 2018.

### Research on Factors Associated With Student Performance

Although the study of variables associated with student performance has historically been a concern in educational research, the publication of the Coleman (1966), together with the following discussion about the central role of socioeconomic variables and the relevance of school practices and policies, started a research line whose relevance has spanned more than five decades and is still highly relevant today. While there are many different sources of data to conduct studies on the variables related to student performance, large-scale assessments have established themselves as a valuable source due to the large volume of variables and observations that they offer to researchers.

Educational variables have traditionally been classified as input and output, later expanded to context-input-process-output, likening the educational process to economic models (Scheerens and Bosker, 1997). However, more recently some authors have suggested to rearrange these categories to better fit ILSA structures, instead choosing to focus around content areas such as school and student background, teaching and learning processes, school policies and education governance, and education outcomes (Kuger and Klieme, 2016). Thus, this section will provide an overview of the scientific evidence of the relevance of PISA variables in relation to secondary education student achievement, following the latter categorisation.

Student context factors are among the most widely studied variables in achievement research. Factors such as socioeconomic status (SES), immigration status, age/grade, attendance to early childhood education (ISCED 0), or grade repetition have been consistently proven to be highly related to student performance (Karakolidis et al., 2016; Pholphirul, 2016; Gamazo et al., 2018). Gender constitutes a special case within this category, since its influence can favor male or female students depending on the competence under study (generally boys outperform girls in math and science, and the opposite is true for reading), also with varying degrees of intensity (Gamazo et al., 2018). At school level, one of the only factors that seem to generate consensus about its positive relationship with performance is mean SES (Asensio-Muñoz et al., 2018; Gamazo et al., 2018). Other school variables, like ownership, resources, student–teacher ratio, or size, have yielded diverse results. There are studies that find no significant relationship between these variables and student performance (Gamazo et al., 2018), some that find positive relationships between school size, resources or ratio and performance (Kim and Law, 2012; Tourón et al., 2018) and others with contradictory results, depending on the country and the PISA wave analyzed. Ownership, for example, has yielded significant results both in favor of public (Kim and Law, 2012; Chaparro Caso López and Gamazo, 2020) and private schools (Acosta and Hsu, 2014). Although the aggregation bias is a widely studied effect (Fertig and Wright, 2005), several studies based on a multivariate methodological framework have aggregated student data to estimate school indices (Brunello and Rocco, 2013; Gamazo et al., 2018; Avvisati, 2020).

Lastly, although ILSAs do not gather information at system level, some studies incorporate these kinds of factors when comparing several countries, and it has been found that background variables related to a country’s affluence and quality of life, like GDP per capita or the Human Development Index (HDI), are closely related to student performance (Täht et al., 2014; Rodríguez-Santero and Gil-Flores, 2018). However, the inclusion of country level variables is relatively uncommon in the literature.

The category of teaching and learning processes encompasses both student and school level variables related to school climate, teaching methodologies, learning time in and out of the school or teacher support (Kuger and Klieme, 2016). While there seems to be some consensus on the positive relationship between student performance and process variables such as climate, learning time or teacher support (Lazarević and Orlić, 2018; Tourón et al., 2018; She et al., 2019), the study of other factors, like inquiry-based teaching practices, yields mixed results (Gil-Flores and García-Gómez, 2017; Tourón et al., 2018).

School policies and educational governance are a less-studied field within large-scale assessment research, although there is some evidence on the positive effect on student achievement of variables like educational leadership, teacher participation in decision-making processes, parental involvement or school autonomy (Drent et al., 2013; Cordero-Ferrera, 2015; Rodríguez-Santero and Gil-Flores, 2018; Tourón et al., 2018).

The last category of variables according to their content area is education outcomes. Student achievement is not the only outcome that education systems should be striving to improve; on the contrary, non-cognitive outcomes like motivation, metacognitive strategies, self-efficacy or domain-related beliefs (Farrington et al., 2012; Khine and Areepattamannil, 2016) constitute a fundamental element when assessing the quality of education systems (Kyriakides and Creemers, 2008; OECD, 2019). Non-cognitive outcomes are usually studied alongside cognitive results, with authors intending to discover the possible relationships between the two kinds of variables. Some of these factors, such as self-efficacy, motivation toward achievement or task mastery, expected occupational status or domain enjoyment have been found to be positively related to student performance (Aksu and Güzeller, 2016; Tourón et al., 2018; She et al., 2019). Metacognitive strategies like summarizing, understanding and remembering, or assessing information have also been positively associated with the students’ reading skills (Cheung et al., 2014, 2016), and they are, in fact, an integral part in some theoretical models that aim to explain student performance through its associated factors, such as the one proposed by Farrington et al. (2012). There are some other variables that have been proven to have a negative effect on student achievement. Such is the case of truancy, which is linked to low levels of achievement, and this relationship is especially relevant in students with low SES (Rutkowski et al., 2017).

Given that the studies reviewed in this section use diverse research methods and include a great variety of different variables, it is not possible to confidently gauge which variables are more relevant overall or have more impact on student performance; instead, only the statistical significance and sign of the relationship (positive of negative) can be reported here.

### Educational Data Mining

Educational data mining (EDM) constitutes an analytical process that enables researchers to turn large amounts of raw data into useful information about different aspects of educational policies and practices (Romero and Ventura, 2010). Although some previous works exist, the main development of this discipline occurred in the first decade of the 21st century, when most of the international conferences and workshops on the subject were first celebrated, and its use has kept on growing in popularity over the past decade (Romero et al., 2010; Tufan and Yildirim, 2018).

Educational data mining is not a method in itself, but rather a group of techniques that share some similarities in terms of procedures and goals. Although there are many different approaches that fall within the scope of data-driven educational research, the main ones, according to their goal, are prediction, relationship mining, and structure discovery (Baker and Inventado, 2014).

The main aim of the prediction approach is to help researchers infer information about a certain variable of interest from a set of other variables (predictors), and also to explore which constructs in a dataset have a relevant role in predicting another (Baker and Inventado, 2014). Prediction can be achieved through two types of techniques: classification and regression, depending on the nature of the predicted variable (categorical or continuous, respectively).

Relationship mining aims to find the strongest relationships among variables in datasets with large amounts of data without a prior designation of criterion or predictor variables. This can be done through different techniques such as association rules or correlation mining (Baker, 2010).

Lastly, structure discovery methods are employed to find natural groupings between data points or variables without a *priori* assumptions of what the analysis should find. The main techniques within this approach are clustering and factor analysis, which look to group together data points/variables that are more similar to those on their group than to those on other groups (Baker and Inventado, 2014).

As we already pointed out initially, EDM-based approaches present some differences from the use of more traditional statistical analysis methods which can be useful in the study of factors linked with performance in large-scale assessments. In this sense, EDM algorithms are being considered by some authors as a more effective and reliable alternative in many aspects than classic inferential and multivariate statistics for the analysis of massive databases (Martínez-Abad, 2019). Moreover, data mining enables the collection of non-trivial information in massive data sets without starting from pre-established models, with minimal human intervention and without raising previous assumptions about the distribution of the data (Xu, 2005).

### EDM and Large-Scale Assessments

Educational data mining can be used to study many characteristics of the teaching-learning process, such as student behavior and/or performance, dropout and retention rates, feedback provided to students, or teacher and student reflection and awareness of the learning process (Papamitsiou and Economides, 2014). Within the field of performance prediction, most of the studies found are conducted at a higher education level, and in virtual learning environments, MOOCs or computer-based learning (Papamitsiou and Economides, 2014). A plausible reason for this is that it is easier to gather large amounts of data from online or computer-based courses given that they allow for the registration of all kinds of participation and interaction data, and these courses are more frequent in Higher Education than in School Education levels.

However, large-scale assessments conducted at a Secondary Education level, such as PISA or TIMSS eighth grade, provide a great opportunity to apply EDM approaches with a less-explored student population. Although the PISA assessment contains static, limited, and structured data, Andreas Schleicher (2013), Director of Education of the OECD and coordinator of PISA, did not hesitate in considering these assessments as big data in education. Other authors have made similar statements, considering the OECD as one of the main providers of system-level big data in the field of education (Sahlberg, 2017).

The use of EDM approaches with large scale assessments is usually focused on predicting student performance in one or more competences (math, reading, and science) by using a set of predicting variables such as student and school background, educational practices or non-cognitive student outcomes, in order to find out which of these variables are more strongly related to performance and thus can serve as better predictors. The past decade has seen the publication of many research works that use EDM techniques for performance prediction. Although there is some diversity in terms of the particular techniques used, the most popular seem to be decision trees and their different algorithms, such as Classification and Regression Trees (CART) (Asensio-Muñoz et al., 2018; Gabriel et al., 2018; She et al., 2019), Chi-squared Automatic Interaction Detection (CHAID) (Aksu and Güzeller, 2016; Asensio-Muñoz et al., 2018; Tourón et al., 2018) or other algorithms like C4.5 (Liu and Ruiz, 2008; Oskouei and Askari, 2014; Martínez-Abad, 2019) or J48, which is another form of the C4.5 algorithm (Aksu and Güzeller, 2016; Martínez-Abad and Chaparro-Caso-López, 2016; Kılıç Depren et al., 2017; Martínez-Abad et al., 2020). Some of these studies aggregate student variables to school level (e.g., Martínez-Abad, 2019), however, there are not, to our knowledge, any basic studies on the effects of the aggregation bias on the computation of data mining models. Another common technique when dealing with student performance data is clustering. This process is usually used to find out which is the best way to group students, schools, or countries according to the similarities in their performance levels, often aiming to conduct a subsequent prediction analysis with said clusters as a criterion variable (Rodríguez-Santero and Gil-Flores, 2018; Soh, 2019). It is worth noting that all the aforementioned studies are focused on single-country analyses.

In this paper, clustering techniques (*k*-means) are used first in order to group schools from 78 countries according to their mean performance level in PISA 2018, and then a prediction analysis is performed in order to discover which country, school, and student variables better predict school performance.

## Materials and Methods

From a purely quantitative approach (Johnson et al., 2007), the main objective of this study is to analyze factors linked to academic performance in large-scale assessments mainly using data mining techniques (Witten et al., 2016), specifically decision trees. To address this goal, secondary data analyses were conducted with PISA 2018 databases (OECD, 2019), where decision trees (C4.5 algorithm) were built to obtain a predictive model of school performance. In this sense, in order to get an integrated and comprehensive model, student and teacher data were aggregated in the schools’ database. In addition, some socio-economic and educational variables at the country level were added to the final database.

Thus, this study follows a non-experimental design based on transversal data (secondary panel data from the PISA 2018 assessment).

### Research Questions

In line with the stated goal, this study seeks to answer four main research questions:

• Is it possible to model school performance by using decision trees and obtain acceptable levels of fit? Which type of factors presents the highest explanatory levels: socio-economic country variables, school indicators and factors, or non-cognitive educational outcomes?
• Do country-level socioeconomic indicators have a relevant impact on performance? Which wealth indicators are more relevant: gross or adjusted?
• Which school indicators have a greater contribution to explain performance? Is their impact conditioned by country-level variables?
• What are the non-cognitive educational outcomes with the greatest contribution to explain performance? Is their impact conditioned by country-level variables?

### Participants

The population of this study were 15-year-old students, teachers, and schools from the countries participating in PISA 2018. Thereby, the initial sample of this research was the entire set of schools, teachers and students included in PISA 2018. An initial review of the data revealed that the Spanish and Vietnamese samples did not include the full scores of the 3 main domains assessed in PISA (science, mathematics, and reading), therefore both countries were removed from the final database.

Thus, the sample was composed of 20,663 schools from 78 countries, and the aggregated data of 570,684 15-year-old students and 85,746 secondary education teachers.

### Variables and Instruments

The full study was carried out using the instruments developed by the OECD for the 2018 PISA wave, which can be grouped in two categories according to their content:

• Context questionnaires: In PISA 2018, different context questionnaires were answered by school principals, teachers, students, and their families. Context questionnaires include a set of items with a wide range of sociodemographic, economic, and educational information related to student outcomes (OECD, 2019). Most of the included items are grouped into constructs referring to different issues: school organization and governance, Teaching and learning factors, student and family background, and non-cognitive/metacognitive factors. The scales obtained from these constructs were calculated using two parameter item-response model. Specifically, PISA uses the Generalized Partial Credit Model, appropriate for working with ordinal items (Martínez-Abad et al., 2020).
• Performance tests in reading, mathematics, and science domains: performance tests include an item bank, and each student is presented with only a fraction of those items. To account for this item disparity, Item Response Theory (IRT) techniques were applied to estimate the ability of the students in each domain. Consequently, the PISA 2018 data does not include a point estimate of a student’s ability in each competence, but rather 10 plausible values that account for the variability in scores depending on the different sets of items available.

Therefore, to define a single criterion variable in this study, it was necessary to apply grouping techniques. Specifically, k-means clustering was used to group schools according to their average performance levels in science, mathematics, and reading. Following previous studies (Shulruf et al., 2008; Zhang and Jiao, 2013; Yao et al., 2015), 3 clusters were obtained: low performance, medium performance, and high performance.

We computed 10 different models, one for each of the 10 plausible values (PV) available, obtaining a final criterion variable with 3 groups:

• Low performance: set of schools classified within the low performance cluster in each of the 10 models.
• High performance: set of schools classified within the high performance cluster in each of the 10 models.
• Medium performance: all other schools.

All variables with high levels of missing values (more than 80%) were removed. In this sense, even though PISA 2018 databases included a sample of teachers only in 19 of the 80 participating countries (including Spain), teacher variables were maintained. This decision was made due to the high level of response of the teacher variables in these countries (in all the teacher variables the general level of missing values is less than 80%), to the construction procedure of the decision trees (based on the consecutive division of the sample to build the model) and the handling of missing values in the C4.5 algorithm (which is not based on data imputation of point values, as noted below). Thus, the predictor variables included in the final database were:

• All the derived variables (scales) available in PISA 2018 from the student, teacher, and school questionnaires.
• All the school-level indicators: school and class size; Ownership; % of students with special needs, with low SES and immigrants; % of girls and repeating students; job and academic expectations of students; Language at home; Additional instruction; Students’ SES; Learning time at school; Attendance to ISCED 0; Average teachers’ age; % of female teachers; Teacher training and development; Teacher employment time; Student–Teacher ratio; Computer-Student ratio.

In addition, the following socioeconomic country indicators were included: Gross Domestic Product (GDP), GDP adjusted by Purchasing Power Parity (PPP), GDP per capita, and GDP (PPP) per capita (International Monetary Fund, 2019); Human Development Index (HDI) (United Nations Development Programme, 2019); and expenditure on education as a percentage of GDP (World Bank, 2020). All the variables included in the study, along with a brief description, can be found in the Appendix.

### Procedure and Data Analysis

According to the technical recommendations (OECD, 2017), school base weights provided in the PISA 2018 database were used in all statistical analyses (student weights were also used when aggregating student variables to school level). After filtering the database, obtaining the criterion variable by using the indicated clustering procedures, and implementing an initial examination of the sample distribution, the decision trees were calculated.

A decision tree includes a set of nested rules, whose graphic representation forms an inverted tree. Decision trees are made up of nodes (which contain the selected predictor variables), branches (which indicate the rules) and leaves (terminal nodes). Thus, trees start with an initial node, which includes the predictor variable with a higher information gain score, and end with a leaf or terminal node, which includes the subsample that complies with all the rules formulated from the initial node to that leaf. Finally, it is important to note that a predictor variable can be included in several tree nodes simultaneously.

The algorithm implemented in the estimation of the final model was C4.5 (Quinlan, 1992). Specifically, we used an extension of C4.5 implemented in the software Weka 3.8 called J48 (Witten et al., 2016). Given its simplicity and characteristics, the use of this algorithm is widespread in Educational Data Mining (Martínez-Abad, 2019). C4.5 and its derived algorithms allow the use of both categorical and numerical predictor variables, and the use of the information gain score (index of the relevance of the predictor variables in a sample that goes through a single branch) to select the predictor variable included in each cut of the tree.

The C4.5 algorithm includes a specific procedure to manage missing data with a probabilistic approach. This approach, which is different from the main imputation methods (e.g., Mean, hot/cold deck, regression, and interpolation), seems to perform better in large databases with a great percentage of missing values (Grzymala-Busse and Hu, 2001), as it is common in large-scale assessments. J48 manages missing data in any predictor variable selected in a node by assigning to each derived branch “a weight proportional to the number of training instances going down that branch, normalized by the total number of training instances” (Witten et al., 2016, p. 230). If another predictor variable with missing values is included in any following nodes of the tree, this procedure is replicated. These instances contribute to the terminal nodes in the same way as the other instances, with their estimated proportional weight.

Initially, we calculated the baseline model, which is quite similar to the null models used in multivariate analysis, since it calculates the fit of a model without predictor variables. Specifically, the baseline model provides the base accuracy level, which is used as a reference to assess the fit of the final model (Witten et al., 2016).

The baseline model was followed by the estimation of the final decision tree that included the predictor variables. In accordance with previous studies (Martínez-Abad and Chaparro-Caso-López, 2016; Martínez-Abad, 2019), the size of the tree was restricted to a maximum of 20 terminal nodes to facilitate the interpretation and to limit the possibility of overfitting of the final model. Although specialized literature recommends the use of a validation procedure in the estimation of the final model (Witten et al., 2016), we included information obtained from both the training set and the 10-folds cross-validation procedure, which facilitates the analysis of overfitting problems. This method implements these consecutive steps (Witten et al., 2016; Martínez-Abad et al., 2020):

• First, the full sample (of size *n*) is divided in 10 approximately equal groups.
• These divisions are used now to obtain pairs of sub-samples. Each pair of sub-samples is composed of both a sub-sample of size *n*/*k* and other sub-sample with the remaining sample, of size *n* − (*n*/*k*). In this process, the 10 possible pairs of different sub-samples are calculated.
• For each pair, the biggest sub-sample will be used as training set (to build the initial model) and the sized *n*/*k* sample will be used as test set (to check the accuracy of the training set model). This procedure will be executed 10 times independently in any of the 10 obtained pairs.
• Finally, the error estimates obtained in all 10 models are averaged to obtain the fit indices and an overall error estimate.

To assess the model fit, the following fit indices were considered (Witten et al., 2016):

• Overall model Accuracy: proportion of the total instances predicted as positive that are correctly classified.
• True Positive rate (TP): proportion of the total number of positive instances that are correctly identified.
• Area under the Receiver Operating Characteristic curve (AUROC): reports on the ability of the model to distinguish between classes. Formally, it can be defined as the probability that the model ranks a randomly chosen positive instance above a randomly chosen negative instance.
• Kappa index: level of agreement between the classification proposed by the model and the true instance classes.
• Root Relative Squared Error (RRSE): proportion of the differences between classes predicted by the model and the true instance classes.

## Results

### *K*-Means Clustering

Table 1 shows the final cluster centers (variable means) in all the computed models. Regardless of the model or the predictor variable, results consistently show high scores in cluster 2, medium scores in cluster 1 and low scores in cluster 3. The contribution of the 3 variables used is highly significant (*p* < 0.001) in all models.

**TABLE 1:** Final cluster centers in 10 *K*-means cluster models.

	Mathematics			Reading			Science		
	Cl 1	Cl 2	Cl 3	Cl 1	Cl 2	Cl 3	Cl 1	Cl 2	Cl 3
PV 1	418.35	517.40	330.93	419.12	518.51	328.85	423.86	518.42	347.23
PV 2	418.29	517.01	332.39	419.50	517.91	329.47	424.34	519.13	347.53
PV 3	418.34	515.84	330.85	419.85	518.43	330.28	424.76	518.55	346.30
PV 4	423.39	518.86	332.96	423.12	520.66	332.60	428.40	521.26	349.69
PV 5	412.66	512.98	329.25	413.46	513.72	325.89	418.86	514.21	345.06
PV 6	415.40	514.83	330.57	415.92	515.48	329.08	421.96	516.25	347.73
PV 7	417.37	517.91	332.48	419.03	518.36	328.87	424.58	519.40	346.85
PV 8	410.72	511.30	328.14	411.14	512.71	325.35	416.60	513.16	345.27
PV 9	416.86	514.54	331.26	415.35	515.97	327.99	420.91	517.30	345.69
PV 10	417.68	516.02	330.60	418.10	516.86	329.55	423.70	518.16	348.15

After obtaining the groups of schools based on clustering, schools were allocated in the following groups: *high performance* (school grouped in cluster 2 in all 10 models), *low performance* (school grouped in cluster 3 in all 10 models) *medium performance* (schools not included in the above groups). The final distribution of schools (Table 2), accounting for the school sample weights, shows approximately 10% more low performance schools than high performance schools.

**TABLE 2:** Final distribution of schools based on clustering models.

	Not weighted		Weighted	
	Freq.	%	Freq.	%
High	7,888	38.17	106,610.51	21.25
Medium	3,087	14.94	157,005.77	31.29
Low	9,688	46.89	238,146.47	47.46
Total	20,663	100.00	501,762.75	100.00

### Input Variables: Country and School Characteristics

All of the country level variables explored showed significant differences when comparing school groups according to performance (Table 3). High performance schools tend to be located in countries with greater levels of GDP (both nominal and adjusted by purchasing power parity and per capita), with greater expenditure on education (% GDP) and greater levels of HDI. The eta-squared (η^2^) effect size scores indicate that HDI and GDP per capita (PPP) are the variables that provide the greatest explanation of the level of performance (in terms of percentage of variance explained). Thus, results show that higher levels of socio-economic wealth, equality and social development promote better levels of academic performance in schools and society.

**TABLE 3:** Country statistics by school performance level.

	GDP*	GDP (PPP)*	GDP pc**	GDP (PPP) pc**	% GDP Ed.	HDI
High	5.110 (7.45)	6.596 (8.47)	30.695 (22.42)	39.951 (17.60)	4.544 (1.21)	0.861 (0.07)
Medium	1.921 (3.94)	3.121 (4.03)	16.147 (16.65)	26.958 (15.39)	4.537 (1.04)	0.800 (0.07)
Low	1.144 (1.79)	2.627 (2.06)	9.027 (8.94)	19.235 (10.96)	4.349 (1.13)	0.755 (0.05)
F (p.)***	26,826 (<0.001)	23,734 (<0.001)	57,205 (<0.00)	63,215 (<0.001)	1,576 (<0.001)	86,610 (<0.001)
D.f.	501,761	501,761	501,761	501,761	498,064	499,703
η^2^	9.660%	8.643%	18.568%	20.126%	0.629%	25.741%

Descriptive statistics and One-Way ANOVA. Rows “High,” “Medium,” and “Low” show mean values, with standard deviation in brackets. *In trillions of dollars; **In thousands of dollars; ***F-statistic (p-value); Total degrees of freedom.

Similarly, school characteristics have a highly significant relationship with school performance (Table 4). While schools with greater average SES and percentage of migrant students are related with high performance, higher proportions of repeating students, together with larger school sizes and teacher-student ratios are related with low performance, with school SES and percentage of repeating students showing the largest effect sizes.

**TABLE 4:** School statistics by performance level.

	SES	SCH size	St-Tch ratio	% immig.	% Repeat.
High	0.097 (0.604)	664.004 (641.448)	12.714 (6.747)	0.095 (0.182)	0.052 (0.089)
Medium	−0.990 (0.935)	431.752 (489.215)	15.463 (12.354)	0.056 (0.149)	0.225 (0.304)
Low	−1.834 (0.746)	333.336 (436.765)	17.465 (13.291)	0.036 (0.124)	0.492 (0.337)
*F* (p.)*	177,969 (<0.001)	12,078 (<0.001)	4,600 (<0.001)	4,854 (<0.001)	79,058 (<0.001)
D.f.	499,488	442,471	436,922	485,053	492,036
η^2^	41.610%	5.177%	2.062%	1.935%	24.584%

Descriptive statistics and One-Way ANOVA. Rows “High,” “Medium,” and “Low” show mean values, with standard deviation in brackets. *F-statistic (p-value); Total degrees of freedom.

Table 5 shows the bivariate distribution by school ownership and performance level. While the distribution of public schools is quite similar in high, medium and low performance schools, private independent and government dependent schools are distributed differently. Although both variables can be considered dependent (χ^2^ = 16,998.42; *p*<0.001), the relationship is weak (Cramér’s *V* = 0.134).

**TABLE 5:** Number and percentage of schools in each performance cluster, by type of ownership.

		Private independent	Priv. Gov. Depend.	Public	Total
High	Freq	18,288	7,775	73,748	99,811
	%	18.3%	7.8%	73.9%	100%
Medium	Freq	35,916	16,691	174,132	226,739
	%	15.8%	7.4%	76.8%	100%
Low	Freq	8,436	23,762	115,466	147,664
	%	5.7%	16.1%	78.2%	100%
Total	Freq	62,640	48,228	363,346	474,214
	%	13.2%	10.2%	76.6%	100%

*Data are weighted by school weight.*

### Decision Tree

The size of the computed decision tree was 36 branches and 20 final leaves. Compared with the baseline model, the average fit obtained in the Training set and Cross-Validation models reached good levels (Table 6): increases in both correctly classified instances (20%) and model accuracy (50%) and an almost 20% reduction in relative error. Moreover, considering that the baseline model classified all the schools as medium performance, levels of accuracy of classified instances in high and low performance clusters could be considered highly satisfactory.

**TABLE 6:** Decision tree fit indices.

		TP	Accuracy	AUROC	Kappa	RRSE
Baseline model (ZeroR). average fit		0.479	0.230	0.500	0	100.00%
Training set	High perform.	0.689	0.802	0.904	–	–
	Medium perform.	0.801	0.666	0.759	–	–
	Low perform.	0.502	0.688	0.877	–	–
	Average fit	0.708	0.715	0.830	0.515	80.97%
Cross-Validation	High perform.	0.688	0.786	0.898	–	–
	Medium perform.	0.789	0.656	0.752	–	–
	Low perform.	0.477	0.679	0.870	–	–
	Average fit	0.697	0.704	0.823	0.496	82.06%

*Baseline model, training set, and cross-validation. TP, true positives rate; AUROC, area under the ROC curve; RRSE, root relative squared error.*

Table 7 shows the confusion matrix obtained in both the full training set and Cross-Validated models. It should be noted that, among the incorrectly classified instances in high and low performance schools, a negligible percentage was assigned by the model to schools grouped in the cluster with the opposite performance. While both the training set and cross-validated models showed less than 1% of schools classified as high performance belonged to the low performance group, less than 1.5% of schools classified as low performance belonged to the high performance group. These results reinforce the previous evidence of the goodness of fit of the predictive model.

**TABLE 7:** Confusion matrices in full training set and cross-validated models.

		Classification (decision tree – J48)					
		Training set			Cross-validation		
		High	Medium	Low	High	Medium	Low
Cluster (*k*-means)	High	89,416.58	39,526.6	772.62	89,271.90	39,815.25	628.65
	Medium	21,242.62	149,805.32	15,907.47	23,520.68	147,551.42	15,883.32
	Low	814.04	35,696.62	36,818.12	760.82	37,586.33	34,981.63

**FIGURE 1:**
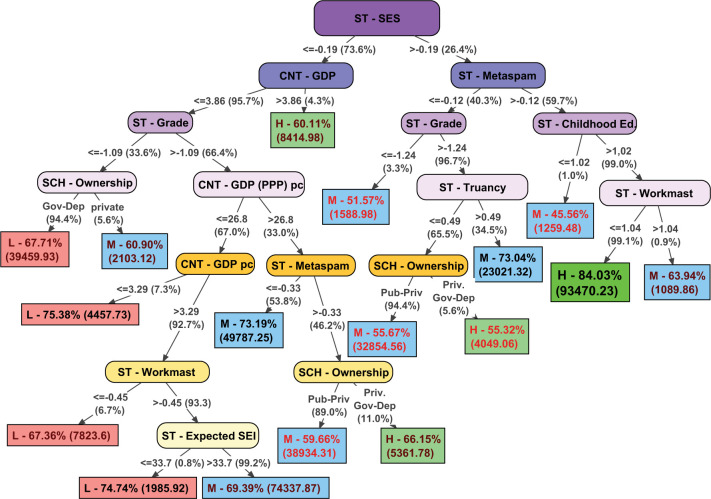
Final decision tree (J48).

The scheme of the model obtained in the decision tree is shown in Figure 1, presenting the following information:

• Oval nodes indicate group segmentation variables. The initial node (ST – SES) performs the first segmentation of the sample, and the different sub-samples go through different branches of the tree, going down it and performing segmentations until reaching a terminal node (leaves).
• The information included in the arrows shows the segmentation score of the sample from the variable of the previous node. For example, for the initial node (ST – SES), the main sample is divided in two sub-samples, one on the left, which includes the instances with scores between (−inf, −0.19], and one on the right with the instances with scores between (−0.19, +inf). The value under parenthesis indicates the percentage of cases of the (sub)sample included in the previous node that progress through that branch.
• The rectangular terminal nodes (final leaves of the tree) include multiple information: first, a capital letter to indicate the group assigned o classification in the predictive model for that sub-sample (L = low performance; M = medium performance; H = high performance); Second, the percentage of correctly classified instances in the sub-sample, highlighting in black the better accuracy (>0.7), in garnet the acceptable ones (0.6–0.7) and in red those of less fit (<0.6); Finally, the numbers in parentheses show the number of instances included in one specific rule or sub-sample.

The first remarkable question that we can observe in the decision tree is the initial node, that is, the first variable of segmentation. The average SES in schools is the variable with a greater predictive power in the model. Taking into account the terminal nodes of the left side of the tree, it can be noted that schools with lower levels of SES are more related to low performance levels. Specifically, in schools with lower levels of SES most of the consequent nodes include socio-economic variables. In this sense, the model indicates that in schools with disadvantaged socio-economic levels the contextual conditions of the country and the school reach a greater importance than in schools with better socio-economic environments.

In the left side of the tree, which tends to show low performance levels, almost only schools located in countries with very high GDP are associated with high performance levels. In this left side, schools with very high grade repetition rates or located in countries with very low per capita GDP are clearly associated with low performance. However, the model includes some non-cognitive educational outcomes that improve the prediction of school performance (ST – Workmast and ST- Expected SEI) in countries with low GDP and per capita income levels. Thus, the job expectations and the culture of effort of the students can be considered factors that promote better academic performance in these disadvantaged schools and contexts. Finally, in countries with better levels of GDP and per capita income, higher levels of student competence to assess the credibility of the information (ST – Metaspam) are related with better school performance levels. Due to the great differences between countries regarding the characteristics of public and private schools, the variable school ownership is hardly interpretable in a single sense.

The right side of the tree is composed of schools with higher average socio-economic levels. The most notable issue in this sub-sample is that the non-cognitive educational outcomes have a greater predictive influence. In this sense, better levels of information credibility assessment in students are clearly related with higher levels of school performance. In fact, the model achieves high accuracy in prediction high performing schools when this factor is combined with not excessively low levels of attendance at early childhood education (ST – Childhood Ed.: more than 1.02 years of attendance to early childhood education on average, a rate reached by more than 99% of schools) and not excessively high levels of self-perceived effort in school tasks (ST – Workmast >1.04, range where more than 99% of schools are located). In schools with lower levels of ST-Metaspam, truancy is the factor with the greatest impact on performance: Schools with non-extreme grade repetition rates in which students, on average, have missed less than 0.49 classes during the last 2 weeks (65.5% of the schools in this sub-sample) are more related to high performance levels.

### Non-cognitive Educational Outcomes

At the educational policy level, the variables of greatest interest are the main non-cognitive educational outcomes included. In this sense, Table 8 shows the distribution of these variables taking into account the main two branches of the tree divided according to school SES. The scores obtained with the full sample indicate low levels of effect sizes in variables Workmast and Expected SEI, moderate effects in Truancy and very high effects in Metaspam. Taking into account the mean scores, schools with low performance have significantly lower levels of students’ Workmast, expected SEI and Metaspam and higher levels of truancy. These descriptive results are quite similar when we divide the sample of schools based on SES. However, this relationship is more intense in the upper SES group of schools, mainly in variables Expected SEI, Metaspam and Truancy.

**TABLE 8:** Distribution of non-cognitive outcomes by School SES and school performance.

		Low SES		High SES		Full sample	
		Mean (*SD*)	F*;η^2^	Mean (*SD*)	F*;η^2^;D.f.	Mean (*SD*)	F*;η^2^;D.f.
Workmast	High P.	0.17 (0.37)	5,438	0.08 (0.37)	1,820	0.10 (0.37)	5,531
	Med. P.	0.16 (0.52)	2.96%	0.08 (0.50)	2.79%	0.14 (0.52)	2.24%
	Low P.	−0.02 (0.51)	420,554	−0.50 (0.95)	62,443	−0.03 (0.52)	483,026
Expect. SEI	High P.	63.7 (11.2)	753	68.6 (7.9)	1,562	67.3 (9.1)	6,737
	Med. P.	63.5 (12.4)	0.42%	65.8 (10.5)	2.39%	64.0 (12.1)	2.71%
	Low P.	62.0 (12.0)	420,595	64.5 (12.8)	62,446	62.0 (12.0)	483,078
Metaspam	High P.	−0.03 (0.42)	33,636	0.17 (0.39)	23,377	0.12 (0.41)	127,357
	Med. P.	−0.50 (0.43)	15.82%	−0.32 (0.41)	26.90%	−0.47 (0.43)	34.43%
	Low P.	−0.70 (0.37)	422,482	−0.55 (0.46)	62,989	−0.70 (0.37)	485,473
Truancy	High P.	0.25 (0.33)	8,561	0.29 (0.27)	5,661	0.28 (0.29)	19,790
	Med. P.	0.49 (0.43)	4.92%	0.50 (0.45)	8.22%	0.49 (0.43)	7.96%
	Low P.	0.61 (0.44)	395,551	0.63 (0.67)	61,833	0.61 (0.44)	457,387

*p < 0.001.

## Discussion and Conclusion

The main goal of this research was to study the different factors comprised in the PISA context questionnaires regarding their ability to predict student performance. The study proposes a systematic process for high and low performing schools through the use of clustering techniques, followed by a predictive approach that yielded results with no interference from previous theoretical models, allowing for the emergence of relationships that might be overlooked or less researched in traditional multivariate literature. In this sense, taking into account the main advantages of Data Mining Techniques (Witten et al., 2016; Martínez-Abad, 2019), it was possible to obtain an explanatory model of school performance based on decision trees with acceptable levels of fit. In contrast to the usual practice in EDM research with large scale assessments (Liu and Whitford, 2011; Oskouei and Askari, 2014; Costa et al., 2017; Kılıç Depren et al., 2017; Asensio-Muñoz et al., 2018; Tourón et al., 2018), and according to current studies (She et al., 2019; Martínez-Abad et al., 2020), we limited the size of the final decision tree. This decision made possible a detailed analysis of the main predictive factors linked with school performance and their interactions. To assess the significance and effect size in the main variables of interest, the information obtained from the decision tree was complemented with descriptive and inferential analyses.

Despite of the small size of the decision tree computed, we achieved levels of fit close to previous studies with less parsimonious models (Liu and Whitford, 2011; Oskouei and Askari, 2014; Costa et al., 2017; Kılıç Depren et al., 2017). These results provide a clear answer to the first research question. In this sense, in line with the findings from previous studies based on multivariate analyses (Täht et al., 2014; Karakolidis et al., 2016; Gamazo et al., 2018), the variables with the greatest impact on the model, located in the initial nodes of the tree, were socio-economic factors both at school and country level.

The most relevant variable to predict school performance is SES, which creates the two main branches in the tree: schools with a mean SES above or below −0.19 (these groups of schools will be referred to as “affluent” and “non-affluent,” respectively). An overall glance at the characteristics of both branches reveals notable differences in the types of variables that appear in each one. The most relevant variables in the affluent schools branch are largely related to educational and individual characteristics, such as metacognitive strategies, ISCED 0 attendance, or truancy. Although these variables have been highlighted by previous multivariate studies (Cheung et al., 2014, 2016; Rutkowski et al., 2017; Gamazo et al., 2018, respectively) the fact that these variables seem to be relevant to student performance only in affluent school settings has not been explored in any of them.

On the other hand, the non-affluent schools branch contains many economic variables such as nominal GDP and its adjusted variants (per capita and Purchasing Power Parity), or school characteristics such as ownership, which appears twice in this branch; student-level educational indicators, such as metacognitive strategies or motivation to master tasks, seem to be less relevant, as they appear nearer the bottom of the tree. All this seems to indicate that, while affluent schools need to turn their focus on improving student-level educational indicators in order to thrive, non-affluent schools’ scores depend in greater measure on economic characteristics that are out of their scope, since they are country-level indicators.

Out of all the country-level economic variables introduced in the model (all of which are located on the non-affluent schools branch of the decision tree), the most relevant one is the country’s GDP without any adjustments per population or purchasing power. This variable generates one of the only two leaves containing high-performance schools in the non-affluent branch, which means that one of the few ways a low-SES school can belong to the high-achievement cluster is by being located on a country with a high nominal GDP; therefore, high levels of GDP (above approximately 4) function as a “protecting factor” for schools with low-SES students. A low level on the other two country variables included (GDP pc and GDP PPP pc) generates a terminal node for low performance schools, leaving little to no space for the consideration of educational variables. Thus, a school from a country with poor economic indicators, both nominal and adjusted, has a meager chance to produce a medium or high level of performance in the PISA test, which attests to the high relevance that economic indicators have as hindering factors for performance (Rodríguez-Santero and Gil-Flores, 2018).

The third research question deals with the school-level variables, of which only school ownership has been included in the model, appearing three times as a node without any derived internal nodes, with medium levels of predictive accuracy. This variable has three possible values, two of which (public and private) have a clear definition in all participating countries. However, the concept of “government-dependent” or “publicly-funded” privately managed schools varies greatly among countries, both in the percentage of public funding allotted and the type of organization managing the school (OECD, 2012). Although this hampers a common interpretation of the meaning and implications of the results of this variable, the positive impact of government-dependent schools on student performance, evidenced by two of the three instances in which it appears in the model, seem to be in line with previous research based on multivariate analyses (Dronkers and Robert, 2008). In any case, these results point to a valuable future line of research that examines the different characteristics and models of government-dependent private schools and their impact on student performance and other outcome variables.

The last research question turns the focus on the educational factors and non-cognitive outcomes included in the decision tree. On the one hand, the relevance achieved by some education indicators in both branches of the tree should be highlighted. In line with previous multivariate studies (Pholphirul, 2016; Gamazo et al., 2018), the model indicates that extremely low average scores in variables Grade and early childhood education attendance prevent schools from belonging to the high-performance cluster. On the other hand, we have previously shown that these variables, mainly non-cognitive outcomes, reach a greater impact in the school performance explanation on the affluent schools branch. We must emphasize that the affluent schools branch includes schools with a high average SES (26.4% of schools sample with higher SES). Thus, in environments with a favorable SES, some educational issues gain relevance. This differential impact depending on the presence of country and school SES has valuable implications for planning educational policies at national levels (Lingard et al., 2013). In this sense, we must study in detail the non-cognitive outcomes included in the model, their contribution and their interactions.

The non-cognitive educational outcome with the greatest contribution to explain school performance has been the students’ competence to assess the credibility of the information. Schools, regardless of having high student SES, can only achieve high performance levels in the model with acceptable levels of fit if their students, on average, reach medium or high skills in information assessment. In fact, the effect size of this variable in the general explanation of the school performance is high, an evidence backed up by other works based on multivariate analyses (Cheung et al., 2014, 2016), adding that these effects are even higher in schools with high SES. Although its effects on the decision tree are weak, school truancy also has a major effect size, mainly in schools with high SES. Bearing in mind that previous studies suggest that the prevalence and effects of truancy are mostly related with impoverished settings (Rutkowski et al., 2017), this result merits further research.

The other non-cognitive factor included in the two main branches of the model is the self-perceived effort in school tasks. Considering that this variable is one of the components of achievement motivation in PISA 2018 (OECD, 2019), it is only logical that a high motivation to master tasks should be related to higher levels or school performance, which is the case in this study and others that have examined achievement motivation and its relationship with performance through data mining techniques (Tourón et al., 2018; She et al., 2019). Finally, in accordance with the previous findings (Tourón et al., 2018), the effects of the students’ expected occupational status are significant, acting as a promoting factor of school performance, especially in low SES schools from low GDP countries, which is a relevant evidence of the importance of fostering high job and academic expectations among all students.

It is worth noting that, although many of these individual findings find support in studies based both on EDM and multivariate statistics, the use of decision trees allows for an in depth study of the relationships that each of the predictor variables have, not only with the criterion variable, but also with each other (Xu, 2005). This feature generates conclusions such as the importance of country-level economic variables only for low-SES schools, or the higher relevance of truancy or early childhood education in more affluent schools, which are not often found in multivariate studies that focus mainly on the relationship established between each predictor variable and the criterion variable (e.g., Acosta and Hsu, 2014; Karakolidis et al., 2016; Gamazo et al., 2018).

Despite this evidence, which seems robust, it is important to note some important limitations linked both with the use of PISA databases and the methodological approach of this study. On the one hand, the use of cross-sectional data makes it difficult to establish causal relationships (Martínez-Abad et al., 2020). Another notable issue is the variability in the indicators and scales used in different PISA waves (González-Such et al., 2016), which are gradually adapting to socio-educative requirements and trends (López-Rupérez et al., 2019). Thus, the replicability and the development of longitudinal studies are hindered. Another key issue related to the processing of the databases is the categorization of the variable academic performance. Despite the fact that we used clustering techniques to avoid human intervention in the process, and that the decision trees are not based on the covariance matrix to build its models, this categorization implies a loss of information in the criterion variable. In future studies, it would be advisable to test the fit of models with a greater number of categories of the criterion variable.

On the other hand, we have used an EDM approach trying to find patterns in big data and to transfer that knowledge to support the decision making of educational policies. It is important to note that we have aggregated student variables to the school database to build the decision tree. In this sense, previous research shows better model fits in decision trees computed with aggregated data in the school level compared to the use of student level as the unit of analysis (Martínez-Abad, 2019).

Apart from that, although the study of the gross academic performance in educational research is widespread (Kiray et al., 2015; Aksu and Güzeller, 2016; Karakolidis et al., 2016; Martínez-Abad and Chaparro-Caso-López, 2016), this practice has led to an overrepresentation of the socioeconomic factors in the predictive model. In fact, despite the presence of the socioeconomic factors in the initial nodes of the model has allowed to differentiate some contexts, we also cannot forget that the educational ecologies are complex and multiple (Bronfenbrenner, 1979; Martin and Lazendic, 2018), which makes it difficult to generalize the results obtained.

Finally, there are some future lines of work that derive from the results and reflections of this study. First, in order to collect more solid evidence on the factors linked with school performance in diverse educational environments, future works should delve into the study of differential performance, testing different predictive models depending on the different socio-economic and contextual conditions (Cordero-Ferrera, 2015; Tourón et al., 2018). Second, considering the vast amount of studies that perform secondary analyses of PISA data, it would be convenient to produce a thorough systematic review in order to explore the different methodologies employed, research questions posed and evidences on the impact of diverse variables on student performance.

## Data Availability Statement

Publicly available datasets were analyzed in this study. This data can be found here: https://www.oecd.org/pisa/data/2018database/.

## Ethics Statement

Ethical review and approval was not required for the study on human participants in accordance with the local legislation and institutional requirements. Written informed consent to participate in this study was provided by the participants’ legal guardian/next of kin. This study is based on the public databases of the PISA 2018 assessment (OECD). Data collection for OECD-PISA studies is under the responsibility of the governments from the participating countries.

## Author Contributions

FM-A: problem statement, methods and statistical models, and interpretation and discussion of results. AG: conceptual framework, discussion and conclusions, and style and structure review. Both authors contributed to the article and approved the submitted version.

## Conflict of Interest

The authors declare that the research was conducted in the absence of any commercial or financial relationships that could be construed as a potential conflict of interest.

## Appendix

**TABLE A1:** Description and Composition of Variables Used in the Study.

Variable Name	Description
**COUNTRY**	
CNT_GDP	Gross Domestic Product
CNT_GDP_PPP	Gross Domestic Product (Purchasing Power Parity)
CNT_GDP_pc	Gross Domestic Product, per capita
CNT_GDP_PPP_pc	Gross Domestic Product (Purchasing Power Parity), per capita
CNT_Porc_GDP_Ed	Percentage of GPD spent on education
CNT_Porc_GDP_Sec	Percentage of GPD spent on secondary education
CNT_HDI	Human Development Index
**SCHOOL**	
SCH_SC001Q01TA	Size of the town/city where the school is located
SCH_SCHLTYPE	School Ownership
SCH_SC048Q02NA	Percentage of students with special needs
SCH_ SC048Q03NA	Percentage of students from socioeconomically disadvantaged homes
SCH_SCHSIZE	School Size
SCH_SC025Q01NA	Percentage of teaching staff attending professional development courses during the last 3 months
SCH_053 (Q01-04, Q09-16)	School offer of extracurricular activities (band, orchestra or choir; school play or musical; school yearbook or newspaper; volunteering; book club; debating club; art club or activities; sporting teams; lectures and/or seminars; collaboration with local libraries; collaboration with local newspapers)
SCH_STRATIO	Student/Teacher ratio
SCH_RATCMP1	Number of available computers per student at modal grade
SCH_RATCMP2	Proportion of available computers that are connected to the Internet
SCH_TOTAT	Total number of all teachers at school
SCH_PROATCE	Index proportion of all teachers fully certified
SCH_PROAT5AB	Index proportion of all teachers ISCED LEVEL 5A Bachelor
SCH_PROAT5AM	Index proportion of all teachers ISCED LEVEL 5A Master
SCH_PROAT6	Index proportion of all teachers ISCED LEVEL 6
SCH_CLSIZE	Class size
SCH_CREACTIV	Creative extra-curricular activities (SC053)
SCH_EDUSHORT	Shortage of educational material (SC017: Q05–Q08)
SCH_STAFFSHORT	Shortage of educational staff (SC017: Q01NA–Q04NA)
SCH_STUBEHA	Student behavior hindering learning (SC061: Q01–Q05, Q11)
SCH_TEACHBEHA	Teacher behavior hindering learning (SC061: Q06–Q10)
**STUDENT**	
ST_GRADE	Grade compared to modal grade in country
ST_ ST004D01T	Gender
ST_AGE	Age
ST_LANGN	Language spoken at home
ST_IMMIG	Immigration status
ST_DURECEC	Duration in early childhood education and care (ISCED 0)
ST_ST062Q01TA	In the last two full weeks of school, how often: I a whole school day.
ST_ST062Q02TA	In the last two full weeks of school, how often: I some classes.
ST_ ST062Q03TA	In the last two full weeks of school, how often: I arrived late for school.
ST_ EC154 (Q01-Q09)	Additional instruction: enrichment or remedial lessons for test language, mathematics, science or foreign language
ST_ECEC	Duration in early childhood education and care
ST_REPEAT	Grade Repetition
ST_BSMJ	Student’s expected occupational status (SEI)
ST_MMINS, LMINS, SMINS	Learning time in mathematics, test language and science
ST_CHANGE	Number of changes in educational biography (Sum)
ST_SES	Index of economic, social and cultural status
ST_UNDREM	Meta-cognition: understanding and remembering (ST164)
ST_METASUM	Meta-cognition: summarizing (ST165)
ST_METASPAM	Meta-cognition: assessing credibility (ST166)
ST_DISCLIMA	Disciplinary climate in test language lessons (ST097)
ST_TEACHSUP	Teacher support in test language lessons (ST100)
ST_DIRINS	Teacher-directed instruction (ST102)
ST_PERFEED	Perceived feedback (ST 104)
ST_EMOSUPS	Parents’ emotional support perceived by student (ST123)
ST_STIMREAD	Teacher’s stimulation of reading engagement perceived by student (ST152)
ST_ADAPTIVITY	Adaptation of instruction (ST212)
ST_TEACHINT	Perceived teacher’s interest (ST213)
ST_JOYREAD	Joy/Like reading (ST160)
ST_SCREADCOMP	Self-concept of reading: perception of competence (ST161: Q01–Q03)
ST_SCREADDIFF	Self-concept of reading: perception of difficulty (ST161: Q06–Q08)
ST_PERCOMP	Perception of competitiveness at school (ST205)
ST_PERCOOP	Perception of cooperation at school (ST206)
ST_ATTLNACT	Attitude toward school: learning activities (ST036)
ST_COMPETE	Competitiveness: dispositional desire to outperform others (ST181)
ST_WORKMAST	Work mastery: dispositional desire to work hard to master tasks (ST182)
ST_GFOFAIL	General fear of failure (ST183)
ST_EUDMO	Eudaemonia: sense of meaning and purpose in life (ST185)
ST_SWBP	Subjective well-being: positive affect (st186)
ST_RESILIENCE	Resilience (ST188)
ST_MASTGOAL	Mastery goal orientation (ST208)
ST_BELONG	Subjective well-being: sense of belonging to school (ST034)
ST_BEINGBULLIED	Student’s experience of being bullied (ST038)
ST_ENTUSE	ICT use outside of school (leisure) (IC008)
ST_HOMESCH	Use of ICT outside of school (for school work activities) (IC010)
ST_USESCH	Use of ICT at school in general (IC011)
ST_INTICT	Interest in ICT (IC013)
ST_COMPICT	Perceived ICT competence (IC014)
ST_AUTICT	Perceived autonomy related to ICT use (IC015)
ST_SOIAICT	ICT as a topic in social interaction (IC016)
ST_ICTCLASS	Subject-related ICT use during lessons (IC150)
ST_ICTOUTSIDE	Subject-related ICT use outside of lessons (IC151)
ST_INFOCAR	Information about careers (EC150)
ST_INFOJOB1	Information about the labor market provided by the school (EC151)
ST_INFOJOB2	Information about the labor market provided outside of school (EC151)
**TEACHER**	
TCH_AGE	Age
TCH_GENDER	Gender
TCH_TC007Q01NA	Year(s) working as a teacher at this school
TCH_TC007Q02NA	Year(s) working as a teacher in total
TCH_TC014Q01HA	Completion of a teacher education or training program
TCH_EMPLTIM	Teacher employment time
TCH_OTT1	Originally trained teacher (strict definition): standard teacher training
TCH_OTT2	Originally trained teacher (wide definition): standard, in-service, or work-based teacher training
TCH_TCSTAFFSHORT	Teacher’s view on staff shortage (TC028)
TCH_TCEDUSHORT	Teacher’s view on educational material shortage (TC028)
TCH_COLT	Test language teacher collaboration (TC031)
TCH_EXCHT	Exchange and co-ordination for teaching (TC046)
TCH_SATJOB	Teacher’s satisfaction with the current job environment (TC198: Q05, Q07, Q09, and Q10)
TCH_SATTEACH	Teacher’s satisfaction with teaching profession (TC198: Q01, Q02, Q04, and Q6)
TCH_SEFFCM	Teacher’s self-efficacy in classroom management (TC199)
TCH_SEFFREL	Teacher’s self-efficacy in maintaining positive relations with students (TC199)
TCH_SEFFINS	Teacher’s self-efficacy in instructional settings (TC 199)
TCH_TCOTLCOMP	Opportunity to learn (OTL) aspects of reading comprehension (TC155)
TCH_TCSTIMREAD	Teacher’s stimulation of reading engagement (TC156)
TCH_TCSTRATREAD	Teacher’s initiation of reading strategies (TC157)
TCH_TCICTUSE	Teacher’s use of specific ICT applications (TC169)
TCH_TCDISCLIMA	Disciplinary climate in test language lessons (TC170)
TCH_TCDIRINS	Direct teacher’s instruction (TC171)
TCH_FEEDBACK	Feedback provided by the teachers (TC192)
TCH_ADAPTINSTR	Student assessment/use (adaption of instruction) (TC202: Q01–Q04)
TCH_FEEDBINSTR	Feedback provided by the teachers (TC202: Q05–Q09)

*The code in brackets indicates which items compose each of the variables used in this study. More information is available in chapter 16 of the PISA 2018 Technical Report (https://www.oecd.org/pisa/data/pisa2018technicalreport/PISA2018_Technical-Report-Chapter-16-Background-Questionnaires.pdf).*
